# 3D‐Printed Metacarpal Prosthesis in the Treatment of Primary Osteosarcoma of the First Metacarpal: A Novel Surgical Technique

**DOI:** 10.1111/os.14282

**Published:** 2024-11-20

**Authors:** Xuanhong He, Leilei Tian, Chang Zou, Minxun Lu, Zhuangzhuang Li, Guy Romeo Kenmegne, Yitian Wang, Yi Luo, Yong Zhou, Li Min, Chongqi Tu

**Affiliations:** ^1^ Department of Orthopedics, West China Hospital Orthopedic Research Institute, Sichuan University Chengdu Sichuan China; ^2^ Department of Model Worker and Innovative Craftsman, West China Hospital Sichuan University Chengdu Sichuan China; ^3^ Department of Anesthesiology, West China Hospital West China School of Nursing, Sichuan University Chengdu Sichuan China

**Keywords:** metacarpal, osteosarcoma, surgical technique, three‐dimensional (3D)‐printed prosthesis

## Abstract

**Objective:**

Osteosarcoma at the first metacarpal is extremely rare. Reconstructing the metacarpal after tumor resection is essential, as the thumb accounts for approximately 40%–50% of hand function. Although autografts, arthroplasty, and transposition have been reported as reconstruction options, their use is limited by complications such as secondary injury, nonunion, and displacement. In this study, we present a case of a patient with first metacarpal osteosarcoma who underwent tumor resection followed by reconstruction with a 3D‐printed metacarpal prosthesis. We tend to introduce a novel strategy to reconstruct the first metacarpal and restore the hand function.

**Methods:**

A 30‐year‐old male with 5‐month history of first metacarpal swelling in the left hand was admitted to our center. Imaging examinations and incision biopsy confirmed a diagnosis of intramedullary well‐differentiated osteosarcoma. A 3D‐printed metacarpal prosthesis was then designed to achieve carpometacarpal (CMC) joint fusion and thumb metacarpophalangeal (MCP) joint reconstruction. Postoperative evaluations included X‐ray and tomosynthesis‐shimadzu metal artifact reduction technology (T‐SMART) imaging to assess bone‐prosthesis integration. Hand function was measured using the Musculoskeletal Tumor Society (MSTS) score and the Disabilities of the Arm, Shoulder, and Hand (DASH) score.

**Results:**

The tumor was completely resected, and a 3D‐printed metacarpal prosthesis was performed to reconstruct the tumor defect. Postoperative imaging showed that the interface between bone and prosthesis was integrated and that there was no loose, displacement, or fracture of the implant. At the last follow‐up, the patient had an MSTS score of 25/30 and a DASH score of 8/100. The range of motion on thumb MCP joint was 30° of flexion and 0° of extension. The Kapandji thumb opposition score was 4 points. The grip strength was 9 kg (compared to 30 kg on the contralateral side) and the key‐pinch strength was 3 kg (compared to 8 kg on the contralateral side).

**Conclusion:**

3D‐printed metacarpal prosthesis could be an effective reconstruction option for patients with low‐grade malignant tumors. Themulti‐planar fixation achieved through 3D surgical planning helps maintain thumb function and restore overall hand function.

## Graphical abstract

In this study, we reported the first metacarpal osteosarcoma patient received tumor resection and 3D‐printed metacarpal prosthesis reconstruction. We tend to introduce a novel strategy to reconstruct the first metacarpal and restore the hand function. During the follow‐up, patients had considerable hand function.

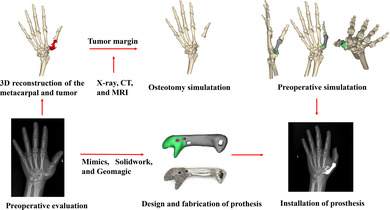

## 1 Introduction

Primary malignant bone tumors in the hand are exceedingly rare, with osteosarcomas in this location accounting for only 0.18%–1.6% of all osteosarcomas [1, 2, 3]. The metacarpal heads and the proximal phalanges are the frequent predilection sites of osteosarcoma in the hand [4]. Before the 1990s, amputation was the primary method for achieving wide tumor resection due to the small size of the muscles and limited expandable soft tissue in the hand [1, 2, 3, 5]. However, patients often face substantial functional loss and cosmetic deformities after amputation [6]. In contrast, limb‐salvage procedures have shown no worsening of prognosis in terms of survival compared to amputation during follow‐up [2]. This shift in approach aims to preserve function and appearance while ensuring effective tumor removal.

With advancements in systemic therapy and surgical techniques, more attempts were made to the limb‐salvage procedures, including iliac or fibular autograft, vascularized iliac or fibular autograft, allografts, silastic prosthesis, and digital ray transposition [6, 7, 8, 9, 10]. While those reconstruction options had made significant changes to function and appearance, there were some problems with the above reconstructive surgery, such as nonunion, graft subluxation, displacement of the proximal phalanx, subsidence of the silicone, and emotion difficulty [7, 10, 11, 12]. Moreover, most of the reconstruction methods performed arthrodesis to the carpometacarpal (CMC) joints with implant, which barely provided single‐dimensional fixation due to the limit of the implant shape and the fixation methods [4, 7, 13]. As widely known, the thumb constitutes approximately 40%–50% of the function and covers 80% of the grip activity of the hand [14, 15]. The maintenance of the functional position of hand includes thumb pronation, abduction, and forward push, which involves three‐dimensional fixation of the thumb to improve the hand function [16]. Therefore, reconstructing the first metacarpal in multiple dimensions is essential to maximize limb function preservation.

Recently, three‐dimensional (3D)‐printing technology has been applied in various bone defects and has achieved favorable results [17, 18, 19, 20, 21]. However, as we know, few study about the reconstruction of metacarpal with 3D‐printing technology was reported. According to Thipachart et al., a 3D‐printed titanium first metacarpal prosthesis was used in treating a patient with giant cell tumor to avoid the complications of the donor site and save the autogenous bone grafts [22]. Nevertheless, there were some shortcomings of this prosthesis and it was not suitable for malignant tumors of the first metacarpal. Herein, we present a rare case with osteosarcoma invading the first metacarpal: (i) to put forward a possible reconstruction strategy for the first metacarpal defect after tumor resection; (ii) to evaluate the clinical outcome and limb function of this kind of reconstruction option.

## 2 Patient and Methods

### 2.1 Case Report

**FIGURE 1:**
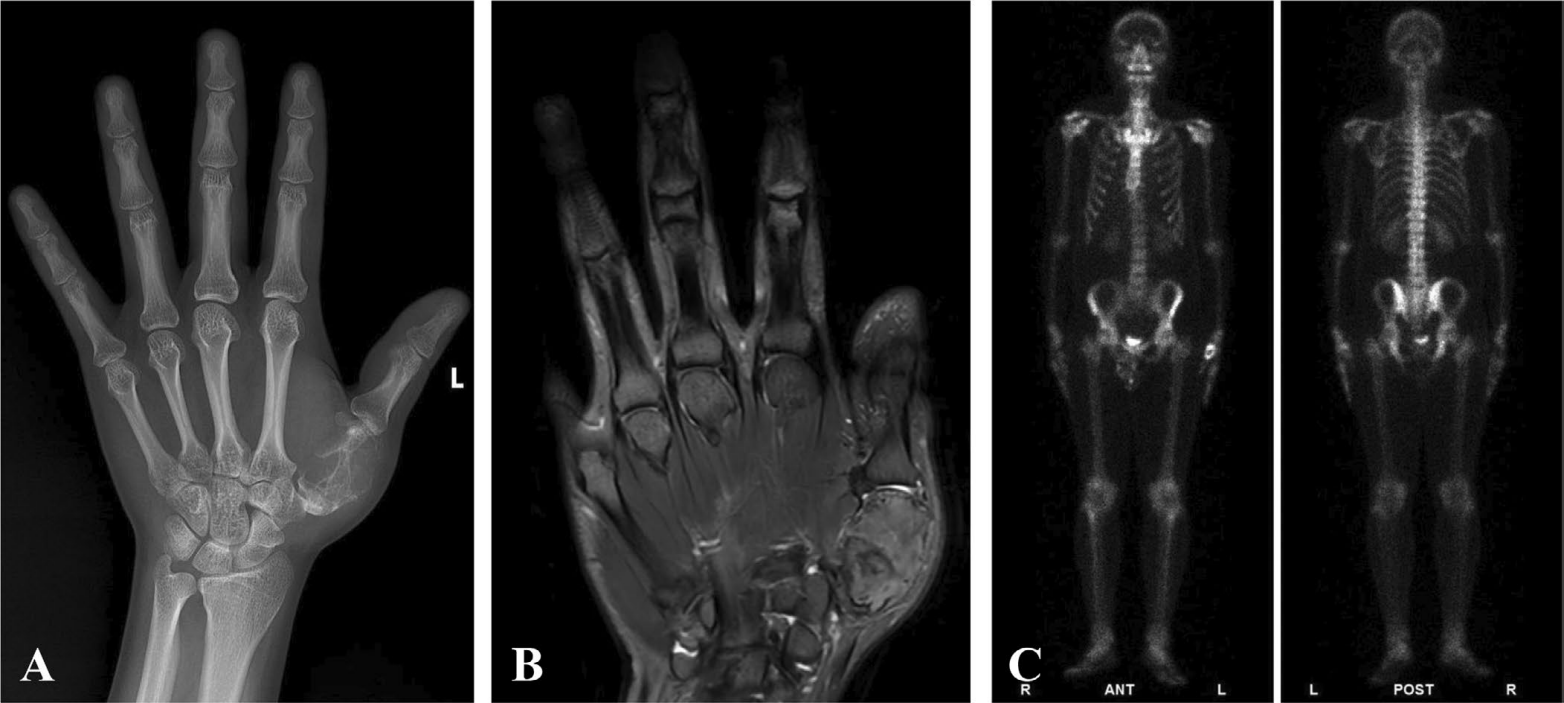
The X‐ray (A) and the MRI (B) demonstrated that the tumor involved the first metacarpal. The SPECT (C) showed no distal metastasis.

A 30‐year‐old male with 5‐month history of first metacarpal swelling in the left hand was admitted to the musculoskeletal tumor center of West China Hospital. The patient reported no relevant trauma or infection, family history of tumor, metabolic, and rheumatic conditions. The physical examination showed that the left first metacarpal was swollen with mild tenderness and a limited range of motion. The Musculoskeletal Tumor Society (MSTS) score was 15/30, and the Disabilities of the Arm, Shoulder, and Hand (DASH) score was 35/100. The Kapandji thumb opposition score is 5 points. The grip strength was 5 kg (30 kg on the contralateral side), and the key‐pinch strength was 1 kg (8 kg on the contralateral side). The anteroposterior and lateral radiograph displayed an osteolytic bone destruction in the first metacarpal (Figure 1A). Magnetic resonance imaging (MRI) showed intramedullary lesion of high signal intensity with clear boundary (Figure 1B). Pulmonary computed tomography (CT) scan and single‐photon emission computed tomography (SPECT) scan indicated no distal metastasis and only the left first metacarpal bone was involved (Figure 1C). Incision biopsy revealed an intramedullary well‐differentiated osteosarcoma. According to the patient's wishes regarding appearance and the evaluation of the tumor boundary, we decided to perform a 3D‐printed metacarpal prosthesis replacement after en bloc tumor resection.

This study was approved by the Ethical Committee of West China Hospital (2022HX04). Written informed consent was acquired from this patient.

### 2.2 Design and Fabrication of Prosthesis

**FIGURE 2:**
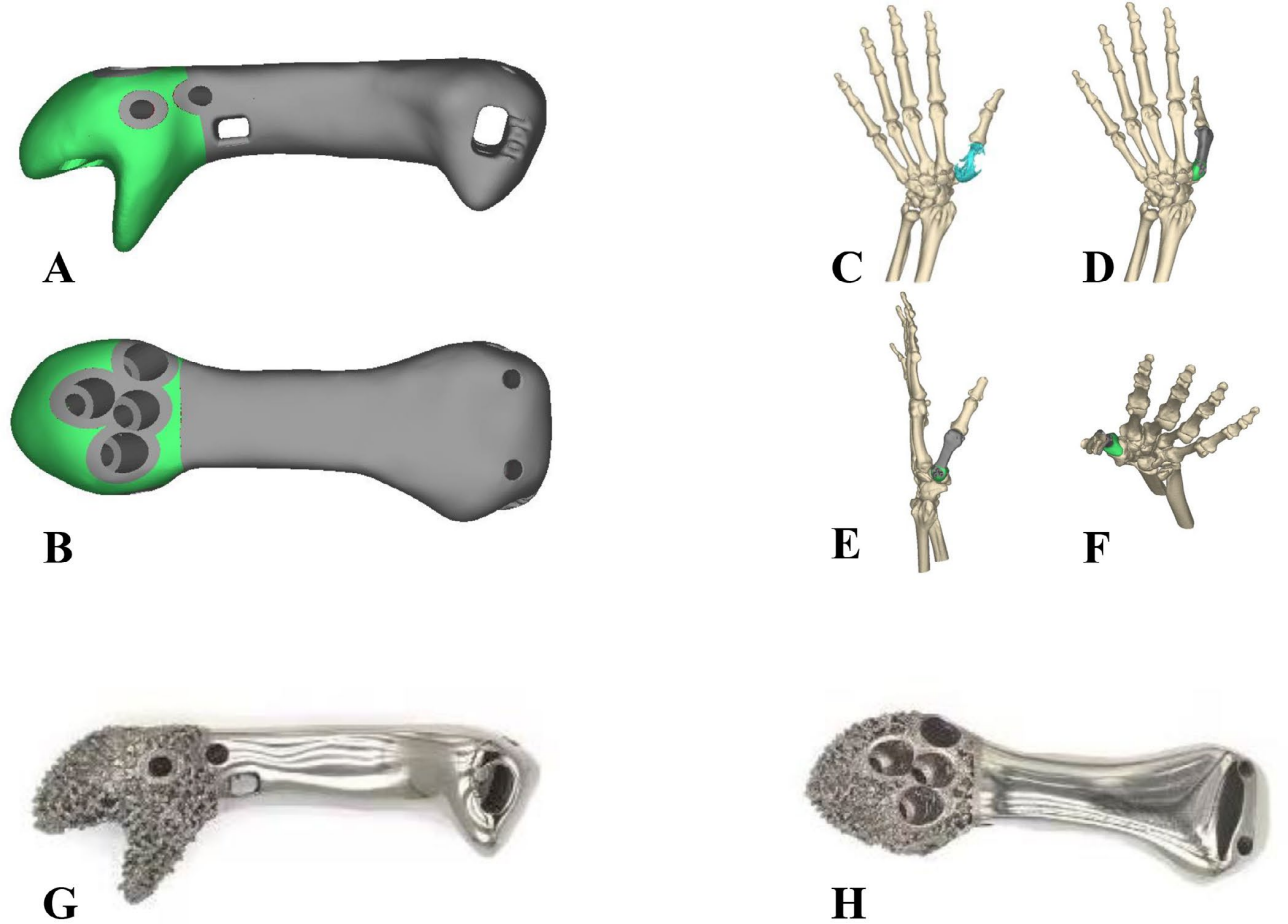
The diagram of prosthesis design (A and B), in which the prosthesis consisted of the proximal porous structure and the distal solid structure. Screw holes and suture holes were designed for joint fixation, ligament, and joint capsule reconstruction. The reconstruction of the tumor defect (C). Preoperative simulation of the fusion position and angle of the CMC joint (D–F). The fabrication of the prosthesis (G and H).

According to the mirror of contralateral images, the metacarpal prosthesis was designed. The prosthesis consisted of the proximal porous structure and the distal solid structure. Screw holes were pre‐set at the proximal to fix the prosthesis with trapezium or scaphoid to realize CMC joint fusion (Figure 2A). Meanwhile, suture holes were designed at the proximal and distal portions of the prosthesis for the reconstruction of the ligament and metacarpophalangeal (MCP) joint capsule (Figure 2A). In order to maintain the functional position of thumb after CMC joint fusion, the prosthesis shape, position, and contact surface between the prosthesis and residual bone were adjusted to ensure the abduction, forward push, and pronation positions of the thumb (Figure 2C). To ensure the consistency of the prosthetic implantation with the preoperative simulation, the following measures are implemented: First, during the preoperative prosthetic design phase, the prosthetic's shape, positioning, and contact surface with the trapezium are adjusted to maintain the first metacarpal in a forward push, pronated, and abducted position. Second, during the operation, it is only necessary to align the prosthetic with the CMC joint surface to ensure the thumb is positioned as simulated preoperatively. Additionally, after the prosthetic implantation, the surgeon verifies the correct positioning of the thumb and compares it against a printed patient‐specific bone defect model and a nylon prosthetic trial model to ensure accurate placement of the implant.

Moreover, the length of bone defect (36.90 mm) was obviously shorter than that in the contralateral metacarpal (47.40 mm). Thus, the length of prosthesis (41.90 mm) was designed to be slightly shorter than that of the contralateral side to reduce the soft tissue tension.

The metacarpal prosthesis was fabricated by ChunLi Co. (Beijing, People's Republic of China). The porous diameter of the prosthesis was 600 μm, and the average porosity was 50%–80% (Figure 2C).

### 2.3 Surgical Technique

**FIGURE 3:**
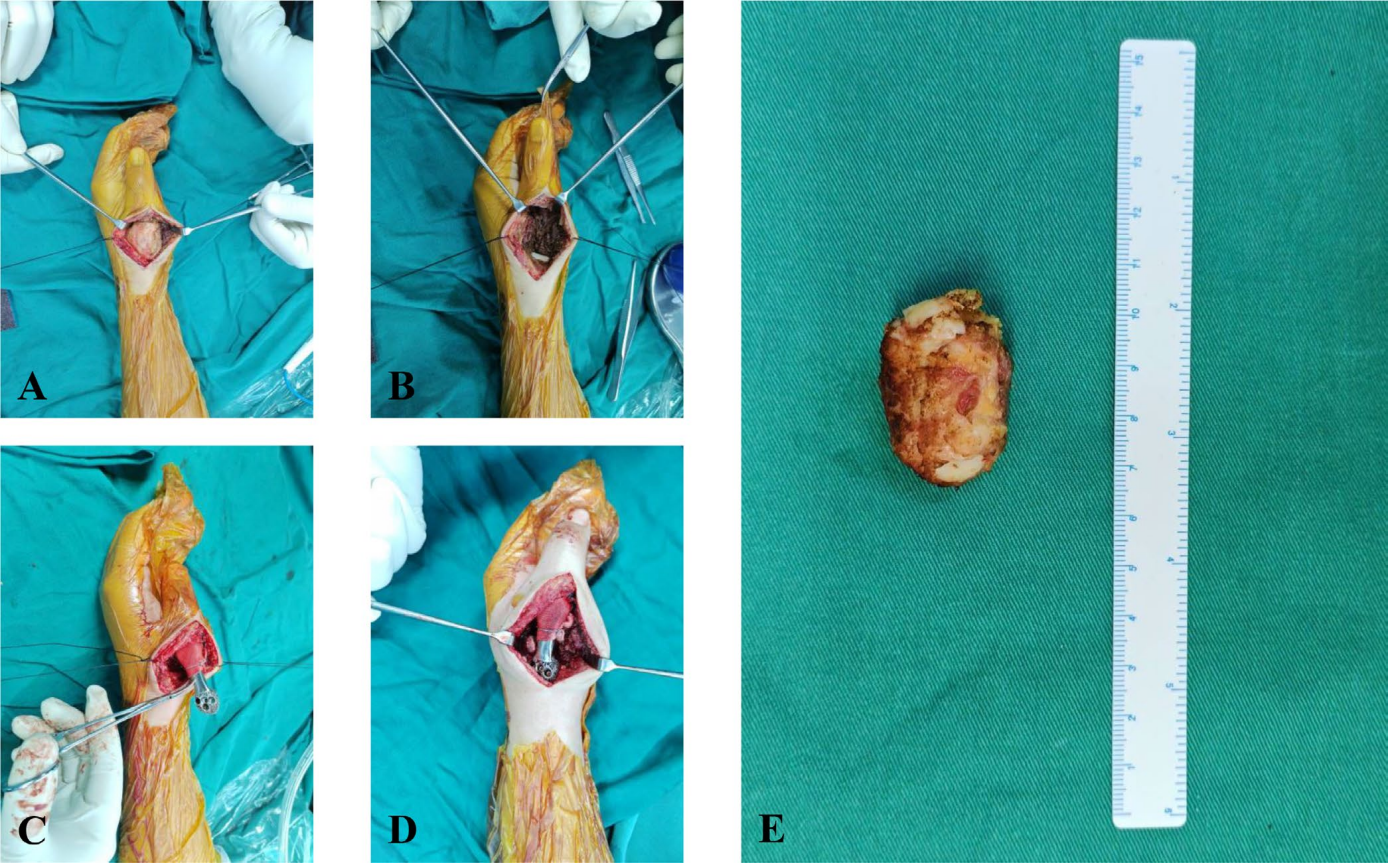
Exposure (A) and complete resection (B) of the tumor. Reconstruction of the MCP joint (C) and fixation of the CMC joint (D). The tumor and the capsule were completely resected (E).

Patient was in the supine position and a tourniquet was placed at the base of the affected limb. A dorsal thumb approach was taken during operation. The superficial branches of radial nerve and artery were protected, and the biopsy channel was removed. As shown, the tumor capsule was intact, and the carpometacarpal joint and the MCP joint were not broken through (Figure 3A). After en bloc tumor resection, the patch was wrapped and sutured at the distal portion of the prosthesis and sutured with the preserved capsule to reconstruct the MCP joint (Figure 3C). Then, the cartilage of the trapezium was removed with a rongeur, and the prosthesis was implanted at the CMC joint and fixed with screws (Figure 3D). As demonstrated in Figure 3E, the tumor was intact resection. Besides, an autogenous iliac bone with a pre‐planned length was implanted between the first and second metacarpals to keep thumb abduction.

### 2.4 Postoperative Management and Follow‐Up

**FIGURE 4:**
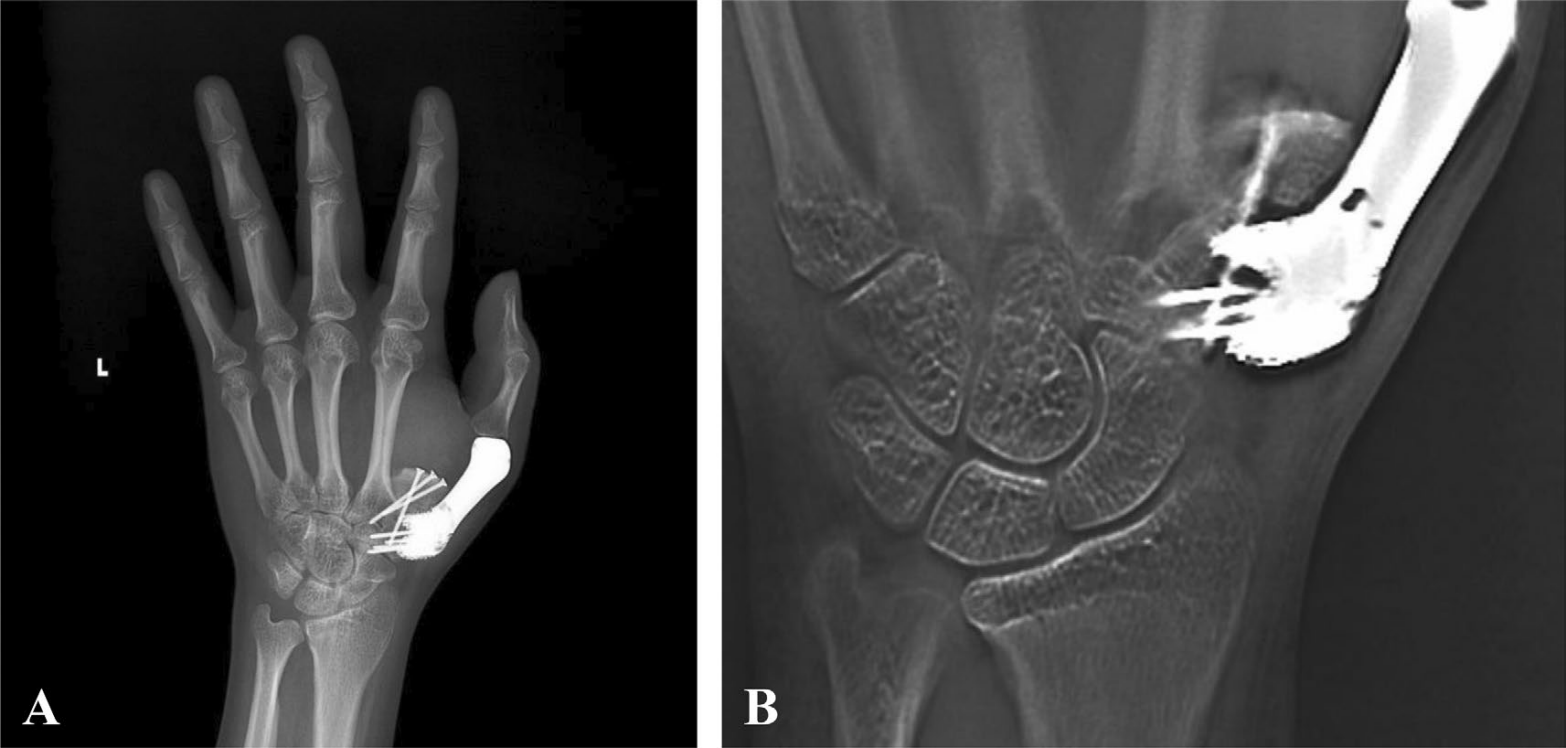
The X‐ray (A) and the T‐SMART (B) of the prosthesis at 6‐month follow‐up. The prosthesis was well‐positioned with no signs of prosthesis or screw loosening. The CMC and MCP joints were well reconstructed. Good osseointegration at the bone‐prosthesis interface was observed.

**FIGURE 5:**
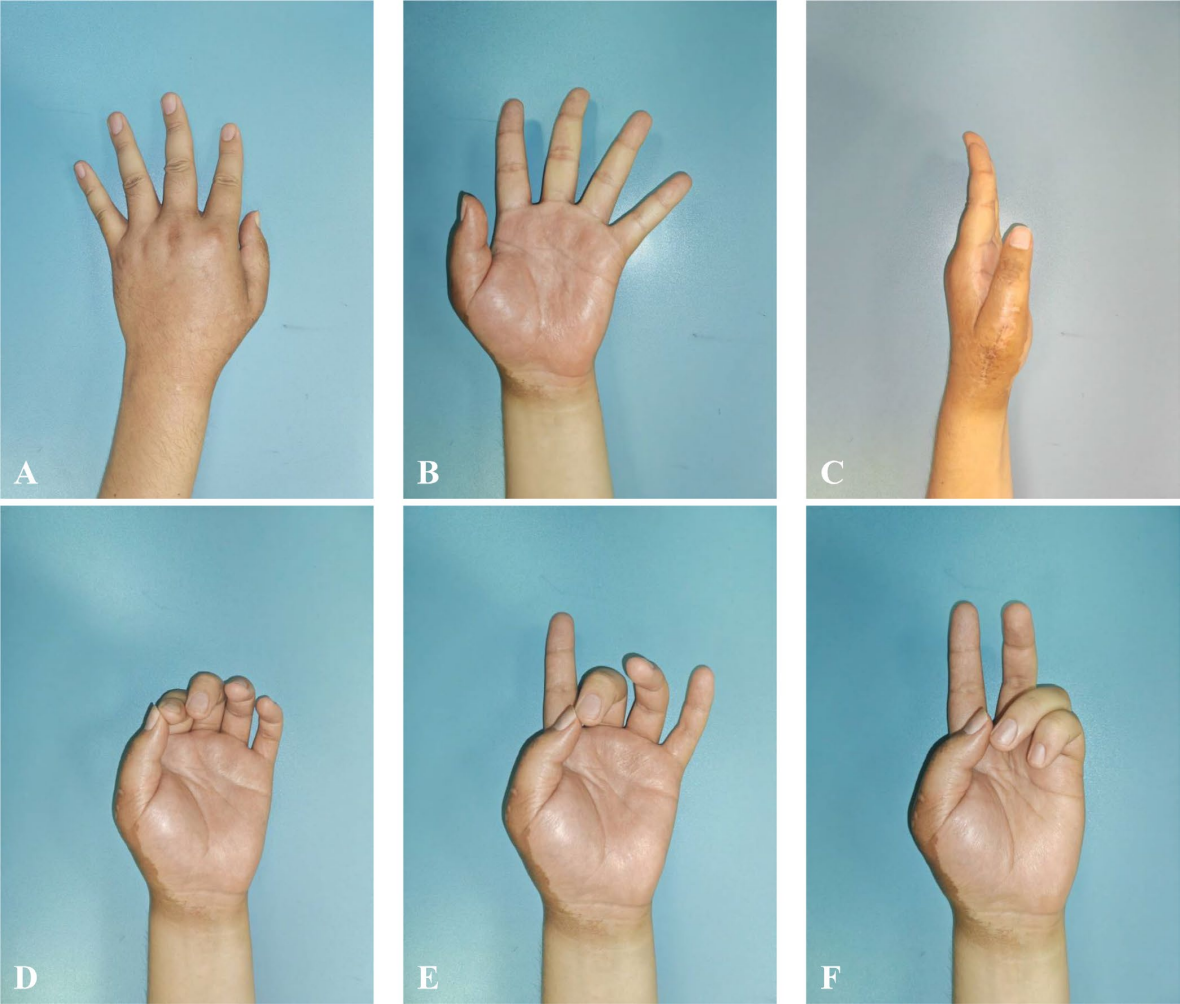
Dorsal (A), palmar (B), and lateral (C) view of the hand. The functional pinch to the index (D), middle (E), and ring finger (F) at 6‐month follow‐up.

The pathological result indicated the diagnosis of intramedullary well‐differentiated osteosarcoma and the margin was clear. Brace immobilization was required for 4 weeks postoperatively. According to the oncology physician consultation, the patient received a radiotherapy schedule of 20 fractions. No complications occurred during follow‐up. During the follow‐up, no local recurrence and distant metastasis were identified. At the time of last follow‐up, the MSTS and DASH scores of this patient were 25/30 and 8/100, respectively. The range of motion on thumb MCP joint was 30° of flexion and 0° of extension. The Kapandji thumb opposition score is 4 points (Figure 4A–F). The grip strength was 9 kg (30 kg on the contralateral side), and the key‐pinch strength was 3 kg (8 kg on the contralateral side). At 6 months postoperatively, the X‐ray and the tomosynthesis‐shimadzu metal artifact reduction technology (T‐SMART) showed that the prosthesis was in a good position without implant displacement or breakage and good integration of bone‐prosthesis interface was observed (Figure 5A,B).

## 3 Discussion

In this study, we introduced a novel reconstruction strategy for the first metacarpal defect caused by osteosarcoma. Considering the rarity of first metacarpal malignant tumors and the challenge of reconstructing the first metacarpal after malignant tumor resection, this study firstly introduces a novel surgical technique to reconstruct the first metacarpal defect and evaluate the clinical effect. With preoperative simulation, the 3D‐printed prosthesis was precisely implanted to restore hand function. Follow‐up results demonstrated favorable hand function and good osseointegration between the bone and prosthesis. Therefore, this study indicates that the 3D‐printed first metacarpal prosthesis could be a potential option for treating malignant tumors of the first metacarpal.

### 3.1 Primary Osteosarcoma in the Metacarpal

The metacarpal is a rare site for primary osteosarcoma [4, 7, 8, 11, 23, 24]. To date, only two cases of first metacarpal osteosarcoma have been reported (Table 1). With advancements in systemic treatment and the improvement of surgical techniques, reconstruction after tumor resection has gradually become available [7, 8, 25]. For benign metacarpal bone tumors, such as GCT, MCP joint, and CMC joint, could be reconstructed after tumor resection to restore hand function to a significant extent [4]. Differently, for patients with malignancy, a wider range of bone and affiliated structures have to be removed in order to achieve a sufficient resection margin. This extensive removal complicates the reconstruction of the MCP and CMC joints, as the muscles and ligaments that provide dynamic function are often resected [4, 23, 24]. Given these challenges, rigid construction of MCP joint or CMC joint may be more suitable for limb salvage in patients with metacarpal malignancies.

**TABLE 1:** Literature review of the osteosarcomas invading the metacarpal bone.

Author	Diagnosis	Location	Reconstruction methods	Number of patients	Follow‐up (months)	Function	Complications
Kyoji et al. 1993	Osteosarcoma	2nd or 3rd metacarpal	Amputation	6	Average 76.7 (15–192)	NM*	4 patients dead and 2 patients alive
Kevin et al. 2006 [8]	Parosteal osteosarcoma	1st metacarpal	Vascularized fibular autograft	1	60	NM	NM
Traske et al. 2007 [11]	Periosteal osteosarcoma	3rd metacarpal	Radial autograft	1	25	No functional limitations	Nonunion
Chen et al. 2023 [24]	Osteosarcoma	2nd metacarpal	Without reconstruction	1	36	NM	Chest wall metastasis
Walid et al. 2021 [4]	Parosteal osteosarcoma	5th metacarpal	No reconstruction	1	36	Average MSTS: 28	NM
Ahmed et al. 2022 [7]	1 low‐grade central osteosarcoma; 1 high‐grade osteosarcoma	1 with 1th metacarpal; 1 with 3rd metacarpal	Nonvascularized fibular autograft	2	Average 66 (24–108)	Average arc of movement: 80° (60°–100°)	Graft subluxation; nonunion

Abbreviation: NM*, no mention.

### 3.2 Reconstruction Options of the Metacarpal Malignant Tumors

As shown in Table 2, the different reconstruction options after metacarpal malignancy resection are reviewed. Autograft, including iliac crest and fibular, was the common reconstruction material. Luis et al. have reported an Ewing sarcoma patient with iliac crest autograft reconstruction of the first metacarpal. During the follow‐up of 108 months, the patient had intact opposition and grasping and could write [6]. Similarly, Atsushi et al. reported the nonvascularized iliac autograft in reconstructing the first metacarpal and the patient presented satisfactory functions during the 37‐month follow‐up [26]. However, graft subluxation and nonunion were the common complications with those nonvascularized autograft or radial autograft, especially the nonvascularized graft with a length greater than 6 cm was difficult to survive as a bone conduction matrix [7, 11, 12]. Vascularized autograft could be an option to improve the union between residual bone and implant. According to Kevin et al., the vascularized fibular autograft was used to reconstruct the first metacarpal in a patient with parosteal osteosarcoma. During the 60‐month follow‐up, no complications, such as graft subluxation and nonunion, were found [11]. Nevertheless, the limited supply, secondary injury, and the extra time to acquire the appropriate graft shape confined the application of autograft in reconstructing the metacarpal.

**TABLE 2:** Malignant tumors rising in the metacarpal and the reconstruction options.

Author	Diagnosis	Location	Reconstruction methods	Number of patients	Follow‐up (months)	Function	Complications
Luis et al. 2013 [6]	Ewing Sarcoma	1st metacarpal	Iliac crest autograft	1	108	Intact opposition and grasping; Patient could write; 45° of motion at the interphalangeal joint	NM
Hills et al. 2013 [9]	Chondrosarcoma	5th metacarpal	Swanson arthroplasty and non‐vascularised iliac crest autograft	1	108	Full range of finger movement	No
Atsushi et al. 2006 [26]	Chondrosarcoma	1st metacarpal	Non‐vascularised iliac autograft	1	37	Satisfactory functions	No
Keiichi et al. 2008 [10]	Chondrosarcoma	4th metacarpal	Digital ray transposition	2	Average 78.5 (12–145)	Average MSTS: 56 (52–60)	Emotional difficulty
						Average DASH: 26	
Sahan et al. 2021 [13]	Enchondroma	1st metacarpal	Non‐vascularised fibular autograft and bio‐tenodesis screw	1	6	Kapandji score 6/10	NM
Walid et al. 2021 [4]	2 GCT; 1 Parosteal osteosarcoma; 1 Ewing's sarcoma; 2 chondrosarcoma	2 with 1st metacarpal; 2 with 2nd metacarpal; 1 with 4th metacarpal; 1 with 5th metacarpal	2 with no reconstruction; 3 with nonvascularized fibular autograft; 1 with extracorporeal freezing; 1 with metacarpal shift	7	Average 52.6 ± 26	Average MSTS: 27.4 ± 1.6	1 with metastasis
Christopher et al. 2016 [28]	1 chondrosarcoma; 1 synovial cell sarcoma	1st metacarpal	Thumb reconstruction with arthrodesis to the second metacarpal	2	Average 36 (24–48)	Average DASH: 15.4 (13.3–17.5)	Wound dehiscence
						Average MSTS: 24.5 (24–25)	
Yves et al. 2003 [29]	Primitive neuroectodermal tumor	1st metacarpal	Digital ray transposition	1	24	DASH: 6.6	

Abbreviations: DASH, Disabilities of the Arm, Shoulder, and Hand; GCT, giant cell tumor; MSTS, Musculoskeletal Tumor Society; NM*, no mention.

Silicone arthroplasty combined with autograft was also applied to reconstruct the MCP joint instead of joint fusion [9, 12, 27]. The application of metal and polyethylene surface replacement components to reconstruct joints could improve the durable result to some extent [9, 12, 27]. According to Hills et al., the Swanson arthroplasty and nonvascularized iliac crest autograft were performed to reconstruct the metacarpal and MCP joint. During the 108‐month follow‐up, the patients had full range of finger movement and no complication was observed [9]. Meanwhile, Edward et al. also reported the Swanson arthroplasty and nonvascularized fibular autograft in two GCT patients, in which the patients had 0°–50° motion at the MCP and no complication [27]. Even so, the use of an implant constrained only by surface congruity may cause postoperative instability owing to the necessary soft loss, especially for patients with malignant tumors. In a patient treated with fibular osteocutaneous free flap and silicone arthroplasty, Neil et al. reported that the displacement of the proximal phalanx and subsidence of the silicone were observed in the follow‐up [12].

Transposition could provide immediate closure of the space after tumor resection and restore hand function with average DASH score from 6.6 to 26 [10, 28, 29]. However, the extended postoperative immobilization to ensure the bone union may cause joint stiffness and extensor tendon adherence. Besides, the potential complications due to the mal‐rotation, mal‐tension of the tendon, and delayed union limited its applications [10]. Moreover, thumb transposition has technical difficulty involving the length, stability, and thumb position reconstruction, in which the tendon transposition may disturb the evaluation of the tumor recurrence [10].

Metacarpal prosthesis was a viable alternative for metacarpal tumors [22, 30]. Thipachart et al. first reported the first metacarpal prosthesis in treating patients with GCT and achieved favorable function outcomes during the follow‐up. Suture holes were distributed in the proximal and distal parts of the prosthesis for ligament reconstruction [22]. Considering that the thumb function accounts for 40% of hand function, the long‐term activity may lead to the displacement of CMC and MCP joints. At the same time, different from benign tumors, malignant tumor resection always involves the peripheral tendons and ligaments, which may cause poor thumb function after nonrigid fixation reconstruction. In addition, for manual workers, rigid fixation of the joint may be more suitable.

### 3.3 3D‐Printed Prosthesis Reconstructs the First Metacarpal Defect

In this case, we designed a 3D‐printed metacarpal prosthesis to reconstruct the first metacarpal defect. Considering the soft tissue defect and the fact that the patient was a manual worker, the CMC joint was designed to be rigidly fixed (Figure 2). The proximal part of the prosthesis was designed as a porous structure to promote bone‐prosthesis integration and make CMC joints fuse stable. During the CMC joint fusion, the limited contact surface between the graft and trapezium may influence the joint fusion [28]. Comparing to autograft or transposition, the 3D‐printed prosthesis may provide more stable prosthesis fixation. The proximal prosthesis was designed with a wider contact interface than the original joint surface with the trapeziums to increase the bone‐prosthesis contact area and improve the probability of bone integration. The temporary stability provided by screws fixation and the long‐term stability provided by the wider porous interface ensures the survival of prosthesis. During the follow‐up, no loosen or fracture of implant was observed (Figure 4). In addition, suture holes were present at the proximal and distal parts of the prosthesis. In this case, the MCP joint was reconstructed through suturing the patch and the reserved joint capsule, which allowed the MCP joint to have a certain range of motion. The motion area of MCP joint in this patient was 0°–30° during the follow‐up. Moreover, the prosthesis should be slightly shorter than the contralateral metacarpal. By reducing the torque and the short lever arm, bone‐implant integration might be improved, and implant failure might be avoided [28]. Besides, the soft tissue defect and tension should also be taken into consideration when planning the length of the prosthesis. Most importantly, due to the particularity of the anatomical position and function of thumb, hand function maintenance relied on the function position of the first metacarpal during the CMC joint fusion. The functional position of first metacarpal includes abduction, forward push, and pronation posture, which involves the fixation of multiple planes. Conventional reconstruction, including autograft or transposition, only fixes the metacarpal through a single plane and does not meet the requirement of hand function reconstruction. As shown in this study, 3D printing technology can adjust the shape of metacarpal according to the functional position of thumb. At the last follow‐up, the patient had a favorable hand function result with 25/30 of MSTS and 8/100 of DASH score. Hand postures in different planes were in accordance with that in the preoperative simulation (Figure 5). Therefore, 3D‐printed metacarpal prosthesis could be an effective strategy to reconstruct the metacarpal after tumor resection.

### 3.4 Surgical Tips

In this study, we introduce a novel reconstruction method for the first metacarpal bone defect caused by osteosarcoma, which is also applicable to other tumors or lesions leading to first metacarpal bone defects. This three‐dimensional reconstruction strategy allows for the reconstruction of the CMC and MCP joints, maximizing the preservation of thumb and hand function. The feature of this reconstruction method lies in the preoperative 3D simulation of the bone defect, along with adjustments in the prosthetic's shape, position, length, and the alignment angles and methods with the CMC and MCP joints, thereby reconstructing the first metacarpal to maintain functional posture. Key techniques of this reconstruction strategy include: first, the prosthetic is advised to be slightly shorter than the contralateral metacarpal to reduce soft tissue tension; second, for patients requiring CMC joint fusion, the proximal end of the prosthetic is suggested to be engaged with more joint surfaces to enhance stability, and it should feature a porous structure to promote osseointegration; additionally, the proximal or distal ends of the prosthetic may be designed with suture holes for the suturing and reconstruction of ligament or the joint capsule. During the operation, the prosthetic reconstruction should be verified against the preoperative simulated angles to ensure the accuracy of thumb positioning and functionality.

### 3.5 Strengths and Limitations

This study was the first to introduce the 3D printing prosthesis in reconstructing the first metacarpal defect after malignancy resection. Considering the rarity of the tumor and the challenge of the reconstruction, this study demonstrated a possible management strategy for the first metacarpal malignancy. Meanwhile, the details of the prosthesis design and the procedure process were described in this study, and the evaluation of the prosthesis stability and hand function were well recorded, demonstrating that this novel technique is feasible for first metacarpal defect. There exists limitation of this study. This is a case report with 6‐month follow‐up. More cases and long‐term follow‐up are needed to evaluate the surgical outcomes and the oncological result of this novel metacarpal prosthesis. Besides, this novel prosthesis could not only be the alternative option for metacarpal reconstruction after malignant tumor resection, but also the benign tumors, osteonecrosis, and traumatic loss of metacarpal.

### 3.6 Conclusion

The 3D‐printed metacarpal prosthesis could be an effective reconstruction option for patients with low‐grade malignant tumors. Multi‐planar metacarpal fixation achieved by 3D operation stimulation contributes to the maintenance of thumb function posture and the restoration of hand function.

## Author Contributions

X.H., L.T., and C.T. designed the research study. L.M. and C.Z. performed the research. Z.L. provided help and advice on revising the manuscript. G.R.K., Y.L., and Y.Z. analyzed the data. X.H., Y.W., and L.M. wrote the manuscript. All authors contributed to editorial changes in the manuscript. All authors read and approved the final manuscript.

## Ethics Statement

This study was approved by the Ethical Committee of West China Hospital (2022HX04). All the patients agreed to participate in this study and signed the written informed consent.

## Conflicts of Interest

The authors declare no conflicts of interest.
